# Correction: Yokoi et al. Erythritol Can Inhibit the Expression of Senescence Molecules in Mouse Gingival Tissues and Human Gingival Fibroblasts. *Nutrients* 2023, *15*, 4050

**DOI:** 10.3390/nu16173041

**Published:** 2024-09-09

**Authors:** Haruna Yokoi, Masae Furukawa, Jingshu Wang, Yu Aoki, Resmi Raju, Yoriko Ikuyo, Mitsuyoshi Yamada, Yosuke Shikama, Kenji Matsushita

**Affiliations:** 1 Department of Oral Disease Research, Geroscience Research Center, National Center for Geriatrics and Gerontology, Obu 474-8511, Japan; haruna.yokoi@ncgg.go.jp (H.Y.); wjsh@ncgg.go.jp (J.W.); karuvachattu@gmail.com (R.R.); yikuyo@ncgg.go.jp (Y.I.); mitsuyos@dpc.agu.ac.jp (M.Y.); shikama@ncgg.go.jp (Y.S.); 2 Department of Geriatric Oral Science, Graduate School of Dentistry, Tohoku University, Sendai 980-8575, Japan; 3 Research Department, Daiichi Sankyo Healthcare Co., Ltd., Tokyo 140-8710, Japan; aoki.yu.g2@daiichisankyo-hc.co.jp; 4 Section of Community Oral Health and Epidemiology, Division of Oral Health, Technology and Epidemiology, Faculty of Dental Science, Kyushu University, Fukuoka 812-8582, Japan; 5 Department of Operative Dentistry, School of Dentistry, Aichi Gakuin University, Nagoya 464-8650, Japan

## Text Correction

Upon review, we have identified an error in the human TNF-α primer sequence reported in Table 1 of our paper titled “Erythritol can inhibit the expression of senescence molecules in mouse gingival tissues and human gingival fibroblasts” [1]. The incorrect sequence was inadvertently included due to an oversight during manuscript preparation. The sequence did not yield successful amplification in our experiments and was not used in the data presented. We provide here the correct primer sequences that were utilized:

TNF-α (F)—CCCAGGGACCTCTCTCTAATC

TNF-α (R)—ATGGCTACAGGCTTGTCACT

The authors state that the scientific conclusions are unaffected. This correction was approved by the Academic Editor. The original publication has also been updated.

The authors thank the readers and the journal for their understanding.
